# EEG alpha band functional connectivity and network structure mark hub overload in Mild Cognitive Impairment during memory maintenance

**DOI:** 10.1192/j.eurpsy.2022.845

**Published:** 2022-09-01

**Authors:** Z. Fodor, A. Horváth, Z. Hidasi, A. Gouw, C. Stam, G. Csukly

**Affiliations:** 1 Semmelweis University, Department Of Psychiatry And Psychotherapy, Budapest, Hungary; 2 National Institute of Clinical Neurosciences, Department Of Neurology, Budapest, Hungary; 3 Amsterdam Neuroscience, Vrije Universiteit Amsterdam, Amsterdam UMC, Department Of Clinical Neurophysiology, Amsterdam, Netherlands; 4 Alzheimer Center Amsterdam, Amsterdam Neuroscience, Vrije Universiteit Amsterdam, Amsterdam UMC, Department Of Neurology, Amsterdam, Netherlands

**Keywords:** Network Analysis, functional connectivity, EEG, mild cognitive impairment

## Abstract

**Introduction:**

While decreased alpha-band functional connectivity (FC) and changes in network topology have been reported in Alzheimer’s disease, it is not yet entirely known whether these differences mark cognitive decline in the early stages of the disease.

**Objectives:**

Our study aimed to analyze EEG FC and network differences in the alpha frequency band during visuospatial memory maintenance between Mild Cognitive Impairment (MCI) patients and healthy elderly with subjective memory complaints.

**Methods:**

FC and network structure of 17 MCI patients and 20 control participants were studied with 128-channel EEG during a visuospatial memory task. FC was measured by amplitude envelope correlation with leakage correction (AEC-c), while network analysis was performed by applying the Minimum Spanning Tree approach.

**Results:**

Increasing memory load enhanced the mean alpha-band FC in the control group. In contrast to that, after an initial increase, the MCI group showed significantly (p<0.05) diminished FC in the highest memory load condition. Mean alpha AEC-c correlated significantly with the size and mean diffusivity of medial temporal lobe structures in the entire sample. The network analysis revealed a rerouted network in the MCI group with a more centralized topology and a more unequal traffic load distribution compared to the control group.

**Conclusions:**

Alpha-band FC correlates with cognitive load-related modulation, with medial temporal lobe atrophy, and with the disruption of hippocampal fiber integrity in the earliest stages of cognitive decline. The more integrated network topology of the MCI group is in line with the “hub overload and failure” framework and might be part of a compensatory mechanism.

**Disclosure:**

No significant relationships.

